# Effect of Transglutaminase Post-Treatment on the Stability and Swelling Behavior of Casein Micro-Particles

**DOI:** 10.3390/ijms231911837

**Published:** 2022-10-05

**Authors:** Ronald Gebhardt, Sahel Khanna, Jann Schulte, Md Asaduzzaman

**Affiliations:** Chair of Soft Matter Process Engineering (AVT.SMP), RWTH Aachen University, 52062 Aachen, Germany

**Keywords:** casein microparticle, cross-linking, stability, swelling, structural model, dynamic model simulation

## Abstract

Casein microparticles are produced by flocculation of casein micelles due to volume exclusion of pectin and subsequent stabilization by film drying. Transglutaminase post-treatment alters their stability, swelling behavior, and internal structure. Untreated particles sediment due to their size and disintegrate completely after the addition of sodium dodecyl sulfate. The fact that transglutaminase-treated microparticles only sediment at comparable rates under these conditions shows that their structural integrity is not lost due to the detergent. Transglutaminase-treated particles reach an equilibrium final size after swelling instead of decomposing completely. By choosing long treatment times, swelling can also be completely suppressed as experiments at pH 11 show. In addition, deswelling effects also occur within the swelling curves, which enhance with increasing transglutaminase treatment time and are ascribed to the elastic network of cross-linked caseins. We propose a structural model for transglutaminase-treated microparticles consisting of a core of uncross-linked and a shell of cross-linked caseins. A dynamic model describes all swelling curves by considering both casein fractions in parallel. The characteristic correlation length of the internal structure of swollen casein microparticles is pH-independent and decreases with increasing transglutaminase treatment time, as observed also for the equilibrium swelling value of uncross-linked caseins.

## 1. Introduction

The interest in developing casein-based microparticles for pharmaceutical or medical applications is steadily increasing due to their biocompatibility and easy availability. To overcome the problems of conventional therapy and enhance efficacy a controlled delivery of the therapeutic agent is essential. There are different approaches in delivering essential drugs and bioactive substances to the target site. For instance, magnetic nanoparticles with different shapes and morphologies, like rod-shape [1], and after coating with organic polymers, usually polyethylene glycol (PEG), can serve to deliver compounds to specific target locations [2]. Another important approach is microencapsulation using biopolymers, which provides protection against harsh environments and facilitates the controlled release of pharmaceuticals. Protein-based biopolymers are suitable for delivering both hydrophilic and hydrophobic bioactive compounds because of their chemical and structural versatility [3]. However, the choice of an appropriate protein for a specific transporter depends on the properties of the polymers (biocompatibility, biodegradability, size, charge, surface properties, etc.), properties of the substance to be encapsulated (solubility, stability, polarity, binding ability, etc.) and properties of the surrounding environment (temperature, pH, ionic strength, solvent properties, etc.). Among proteins that have been tested for delivery systems, milk protein (e.g., casein) has proven an efficient encapsulation material owing to its structural and functional properties [4,5,6]. The milk protein, casein, exhibits an unfolded conformation required to form a thermodynamically stable complex with amorphous calcium phosphate [7]. In their native state, casein micelles are polydisperse, spherical particles with a mean diameter of 160 nm [8]. Their inner structure is highly porous and hydrated [9,10,11].

The typical process of producing microparticles involves gel formation, emulsification, spray drying, and heat treatment [12]. For instance, caseins form gels with polysaccharides (gum, carboxymethyl cellulose, and pectin), which can also be used for encapsulation of bioactive compounds [13,14]. A gentle method for the production of casein microparticles (CMPs) is based on flocculation aggregation of casein micelles under neutral pH conditions at room temperature [15]. In this process, several µm-sized casein aggregates are formed in solution due to the volume exclusion of added pectin [16], but these aggregates disintegrate again when the pectin is removed. However, the casein aggregates can be compacted by film drying in the pectin matrix and solidified after ellipsoidal deformation [15]. These can then be removed from the film matrix by degradation of the pectin using the enzyme pectinase and subsequently resolubilized in a buffer while returning to their spherical shape [17]. 

The investigation of the stability and swelling behavior of CMPs is of interest in view of their potential use as carriers for bioactive compounds for immediate or controlled release application. Stability studies have shown that CMPs completely decompose after the addition of sodium dodecyl sulfate [18], suggesting that hydrophobic interactions are crucial for cohesion [19,20]. In contrast, electrostatic contacts are also crucial for the swelling of CMPs, since water uptake strongly depends on the pH value and calcium ion content of the swelling medium. If they expand to more than twice their microscopically observed projected area during this process, the physical interaction contacts are no longer sufficient and the CMPs dissolve [21]. While swelling is completely inhibited in the acidic and neutral pH range, strong water absorption occurs in the basic pH range. At pH 8, the particles swell and disintegrate only after hours, while this happens within minutes at pH 11 and within seconds at pH 14 [22]. However, the swelling process can also be suppressed at pH 11 if calcium ion concentrations > 10 mM are present [21]. In the basic pH range, caseins are negatively charged and interact with water all the better with increasing pH and charge state. During swelling the polymer strands gain in mixing and configurational entropy—but if a chemically cross-linked network is present, the strands are stretched and configurational entropy decreases [23]. Chemically cross-linked networks such as acrylamide hydrogels therefore generally exhibit lower equilibrium swelling percentages [24]. Caseins can be chemically cross-linked with the help of the enzyme transglutaminase (TGase), which catalyzes the acyl-transfer reaction between the carboxyl group (-COOH) of glutamine residue and the ε-amino group (-NH_2_) of a lysine residue of casein [25,26]. On the one hand, this increases the colloidal stability of the casein micelles and their resistance to various chemical and process conditions [26,27,28,29], and on the other hand, higher aggregated structures can also be stabilized [30,31].

The most common release strategy is achieved by disintegration or swelling of the carrier particles [32]. The ability of CMPs to protect themselves from complete disintegration after swelling is of interest for the controlled release and encapsulation of bioactive compounds. In the latter case, swelling can lead to convective uptake of substances by the unloaded microparticle matrix in addition to diffusive uptake [33].

The use of CMPs has several advantages over nanoparticles. In contrast to CMPs, drastic process condition such as high hydrostatic pressure treatment is needed for production of casein nanoparticles [13]. For use as an oral delivery system, colloidal stability under acidic conditions is required for gastric passage. With their interesting pH-dependent swelling behavior and high acid stability, CMPs exhibit optimal characteristics for this purpose. Native nano-sized casein micelles, however, tend to aggregate and form a gel under these conditions and are hence not stable [34]. Furthermore, casein nano-micelles would simply be too small to encapsulate probiotic microorganisms [35].

Therefore, the aim of this study was to investigate the influence of TGase post-treatment on the swelling behavior of CMPs with the purpose of preventing decomposition. For a detailed investigation of the swelling mechanism, we performed the experiments at pH 11 and pH 14 because under these conditions they ran at experimentally feasible times and the results could be extrapolated to physiologically relevant pH values [22]. The influence of the TGase treatment time and the pH value of the swelling medium on the swelling kinetics was analyzed by means of a dynamic model. Dynamic model simulations are a powerful tool in the field of molecular sciences to understand complex processes such as parallel crystallization and aggregation [36], multistep swelling processes [21], or permeation of drugs during particle dissolution and membrane diffusion [37]. In addition, we investigated the effect of TGase treatment on SDS stability and the internal structure of CMPs.

## 2. Results and Discussion

**Figure 1:**
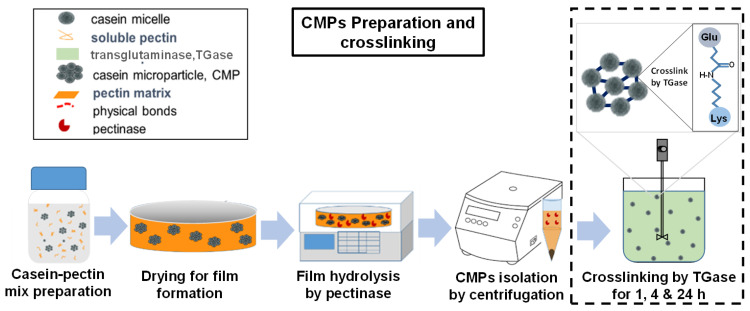
Manufacturing process of CMPs that first leads to aggregates of casein micelles by volume exclusion of the added pectin, which are subsequently solidified by film drying to form CMPs that are then released by degradation of the pectin matrix by pectinase and resolubilized in buffer at pH 6.7. The subsequent cross-linking of the CMPs produced by the enzyme TGase is highlighted.

Figure 1 shows the individual steps of the process for the gentle production of CMPs [15]. For the individual process steps, i.e., the preparation of the casein-pectin mixture, the solidification of the resulting aggregates in CMPs by film drying, and their preparation from the pectin matrix, standard conditions were chosen as described in Section 3.3. For the preparation of the samples for this study, the CMPs were then post-treated with 0.155% (*w*/*w*) TGase under moderate stirring for different times. 

The cohesion and stability of CMPs can be investigated by turbidity measurements using SDS addition. Previous studies have shown that CMPs decompose completely and nearly mono-exponentially after the addition of SDS. This is an indication that CMPs are mainly stabilized by hydrophobic interactions. In contrast, calcium-mediated electrostatic contacts affect the rate of decomposition, as shown by analysis of the turbidity curves of CMPs preparations with added low-esterified pectin [18]. 

**Figure 2:**
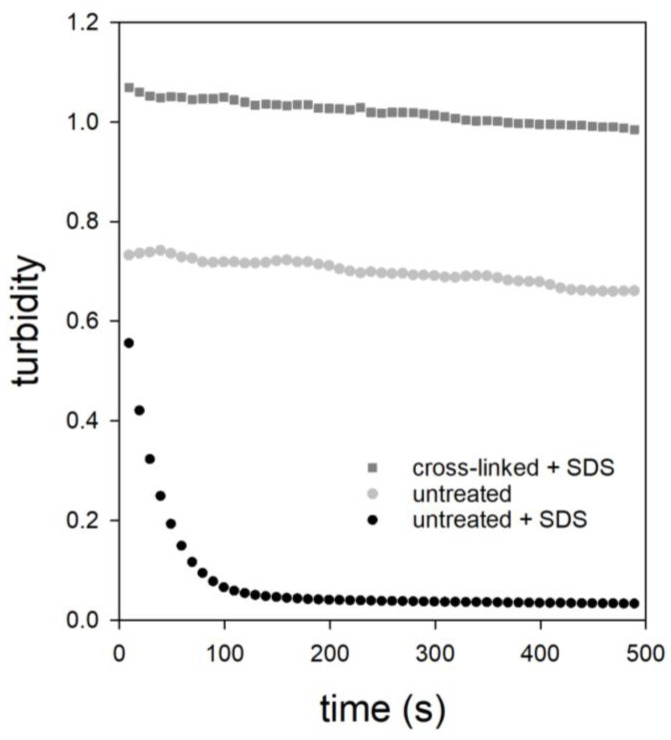
Turbidity as a function of time of CMPs with (dark gray) and without TGase treatment (black) after the addition of SDS. The measured data of a CMPs sample without TGase treatment and SDS addition is also shown (light gray).

We studied the time course of the turbidity of a TGase-treated sample to test the influence of covalent cross-linking on the stability of CMPs. These experiments under non-physiological conditions should provide the first indication of whether TGase cross-linking is enough to ensure the structural integrity of the microparticles. The cross-linked CMPs sample exhibited higher initial turbidity in contrast to the untreated sample, as shown in Figure 2. This could be due to the fact that the protein content in these CMPs is higher because of the covalent cross-linking, which prevents the leakage of individual caseins [38]. Based on our microscopic observations we have no indications that a higher number of aggregated CMPs resulted from TGase-treatment. Furthermore, the turbidity of the cross-linked sample did not decrease mono-exponentially but linearly and to a lesser extent with time. We also studied the turbidity curve of an uncross-linked CMPs sample to which no SDS had been added in order to understand this result. As the comparison shows, this sample had lower turbidity at the beginning probably due to the lack of covalent cross-linking, but similar to the cross-linked sample with SDS, the turbidity decreased only slightly and linearly with time. This decrease was due to the much slower sedimentation to which the CMPs were exposed. Sedimentation depends not only on the diameter and density, but also on the shape of the CMPs. The two comparable linear sedimentation profiles show that TGase-treatment did not result in dramatic changes in the size, density, and shape of the CMPs, but did prevent their SDS-induced decay. The latter is probably a consequence of the covalent linkage of neighboring casein regions, which were previously stabilized only via hydrophobic interactions. 

**Figure 3:**
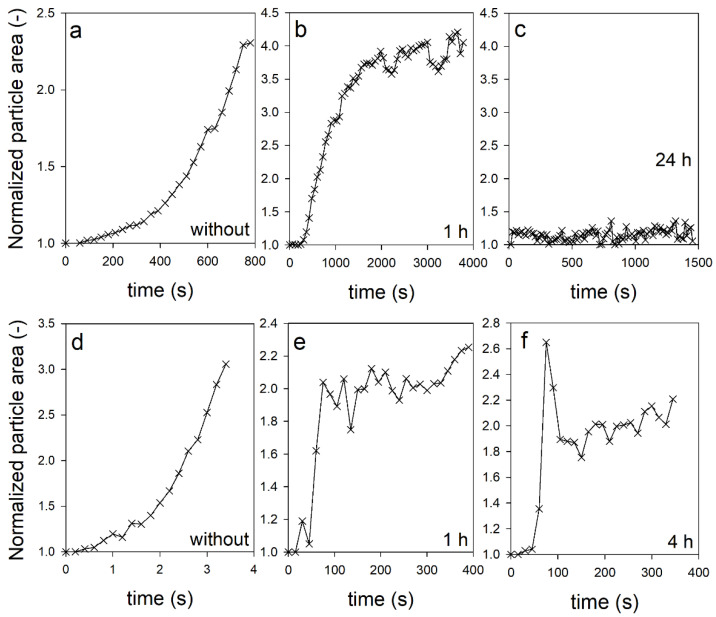
Change in relative particle area with increasing swelling time for CMPs at pH 11 ((**a**) without cross-links, (**b**) cross-linked for 1 h & (**c**) cross-linked for 24 h) and at pH 14 ((**d**) without cross-links, (**e**) cross-linked for 1 h & (**f**) cross-linked for 4 h).

In addition to stability, we investigated the effect of TGase post-treatment on the swelling of individual CMPs at pH 11 and pH 14. This involved the use of sieve cells, which allowed microscopic single-particle observation of swelling in real-time [22]. In previous experiments, we had shown that the microparticles swell in a two-step process and finally disintegrate. Aiming to prevent the complete disintegration of the particles, we treated the CMPs with TGase for different time periods before the swelling experiments. Figure 3 shows the swelling kinetics of the CMPs at pH 11 (a–c) and at pH 14 (d–f). In each case, the results of the untreated CMPs are shown on the left for comparison, the results of the CMPs treated with TGase for one hour are shown in the middle, and the results of the CMPs treated for 24 h and 4 h are shown on the far right. The swelling curve of the untreated samples confirms our earlier observation that the CMPs swell slowly in the first step and then faster in the second step until they finally decay completely. While this takes several minutes at pH 11, at pH 14 swelling and complete disintegration occurred within seconds [22]. At pH 11, a 1 h TGase post-treatment of the CMPs led to a changed swelling curve. The initially steep increase of the particle area decreased more and more with increasing swelling time and finally approached a plateau value after approx. 2000 s. 

However, periodic dips were detected in the swelling data, which occurred approximately every 1000 s and were never previously observed in the swelling curves of untreated CMPs. In contrast, the swelling kinetics of the same CMPs showed some striking differences at pH 14. For example, there was already a low initial swelling at the beginning before the swelling curve rose abruptly after approx. 50 s. After that, it dropped slightly and then continued to rise slowly at swelling times >150 s. This typical profile was even more pronounced for CMPs treated for 4 h with TGase. Under these conditions, the particle area swelled to 2.6 times the initial size at the beginning and reached a maximum value. In the further course, the particle area decreased just as quickly back to twice the initial size, and then from approx. 150 s onwards it showed a course similar to that of CMPs treated with TGase for 1 h only. In contrast, a 24 h treatment of CMPs with TGase led to complete suppression of the swelling process at pH 11.

We used system dynamic modelling to analyze the swelling kinetics. For the analysis of the two-step swelling kinetics of the uncross-linked CMPs, we previously used a sequential model [22]. The individual swelling steps were represented by two volume flows, each of which depended on the present volume of the particle, a rate, and a characteristic time at which the valve opened for the respective volume inflow. When modelling the swelling curves of CMPs treated with TGase, two special features must be taken into account. On the one hand, due to the cross-linking, the particles do not disintegrate after the swelling process but remain stable. On the other hand, the shape of the swelling curves depends on the treatment time with TGase, as observed in the right images compared to the middle images in Figure 3 (top and bottom row). This means that the degree of crosslinking changes with the treatment time and that the CMPs thus consist of one part of uncross-linked caseins and one part of cross-linked caseins [27]. 

**Figure 4:**
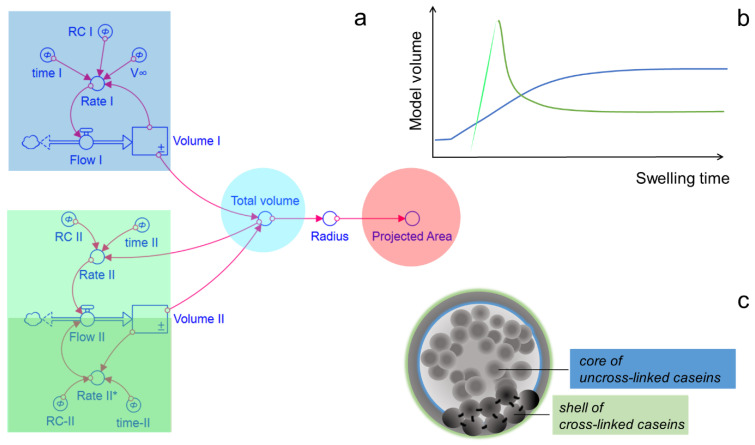
Analysis of the swelling process of CMPs treated with TGase. (**a**) Parallel system dynamics model; (**b**) Time course of the different swelling fractions and (**c**) schematic representation of the CMPs consisting of a core of uncross-linked and a shell of cross-linked caseins (Not true to scale).

Both fractions are considered in the parallel dynamic model shown in Figure 4. Studies on gelled food matrices have shown that cross-linking rates depend on the pore size and the accessibility of the substrates by the enzyme within the gel network [39,40]. Because caseins are most accessible to TGases at the surface, we assumed that cross-linked caseins formed a shell around a core of uncross-linked caseins inside the CMPs (Figure 4c). 

The two casein fractions are assigned to the reservoirs Volume I and Volume II, and the sum of both gives the total volume that simulates the projected particle area taking the spherical approximation into account. Volume I, which describes the swelling state of the CMPs due to the uncross-linked caseins, increases only to a limited extent during swelling (blue curve in Figure 4b). We use second-order swelling kinetics for modelling, which was originally used for the swelling of gelatin and cellulose in the aqueous medium [41], but has recently also been successfully used to describe the swelling of micellar casein fibers [42]. In this approach, the rate of volume change depends on the specific swelling rate, expressed by the rate coefficient I (RC I), and on the square of the remaining swelling capacity $\left(V_{\infty }-Volume\;I\right)$, according to:

(1) $$\frac{dVolume\;I}{dt}=RC\;I\cdot {\left(V_{\infty }-Volume\;I\right)}^{2}$$

Complete decomposition by swelling, as occurs with untreated CMPs, is thus prevented by a decreasing swelling capacity with time, which could be caused by the increasing stress state of the network of cross-linked caseins. The maximum achieved swelling volume of the uncross-linked caseins V_∞_ changes as a function of the TGase treatment time. The corresponding values for V_∞_ obtained by model analysis normalized to the initial volume V_1,0_ are shown in Figure 6b. The maximum swelling volumes reach 2 to 2.2 times the initial volume after one hour of TGase treatment regardless of pH, while for untreated CMPs there is a pH dependence toward larger swelling volumes at higher pH values [22]. This observation suggests that the elongation state of TGase-treated CMP is dominated by the elastic restoring force of the cross-linked caseins and that the charge state of the caseins plays little role. In contrast, the maximum equilibrium degree of swelling is influenced by the treatment time with TGase, since more covalent bonds increase the elasticity of the network. The maximum degree of swelling decreased with increasing TGase treatment time to 1.8 after four hours of treatment at pH 14 or to 1 after 24 h of treatment at pH 11. According to our conception (see schematic Figure 4c), the corresponding network was located at the surface of the CMPs and forms a shell there, with an increasing proportion of the total volume with increasing TGase treatment time. The cross-linked caseins must be responsible for the unusually strong increase at the beginning of swelling and the subsequent deswelling observed at pH 14, particularly pronounced at longer TGase treatment times (see Figure 3). This characteristic part of the swelling curve is shown as a green curve in Figure 4b. The pronounced expansion peak is likely a result of over-expansion, where the network overstretched as a result of swelling and then retracted back to the equilibrium value (green curves in Figure 4b). The dynamic model simulated the influence of the network of cross-linked caseins through volume II. The corresponding volume change rate is composed of a swelling rate (rate II) and an unswelling rate (rate II*) to simulate the expansion and recovery of the network through volume II. While the swelling rate is a function of the rate coefficient II (RC II) and the total volume, the deswelling rate depends on volume II and on a specific deswelling rate expressed by RC II*.

(2) $$\frac{dVolume\;II}{dt}=RC\;II\cdot total\;Volume-RC\;II^{*}\cdot Volume\;II$$

**Figure 5:**
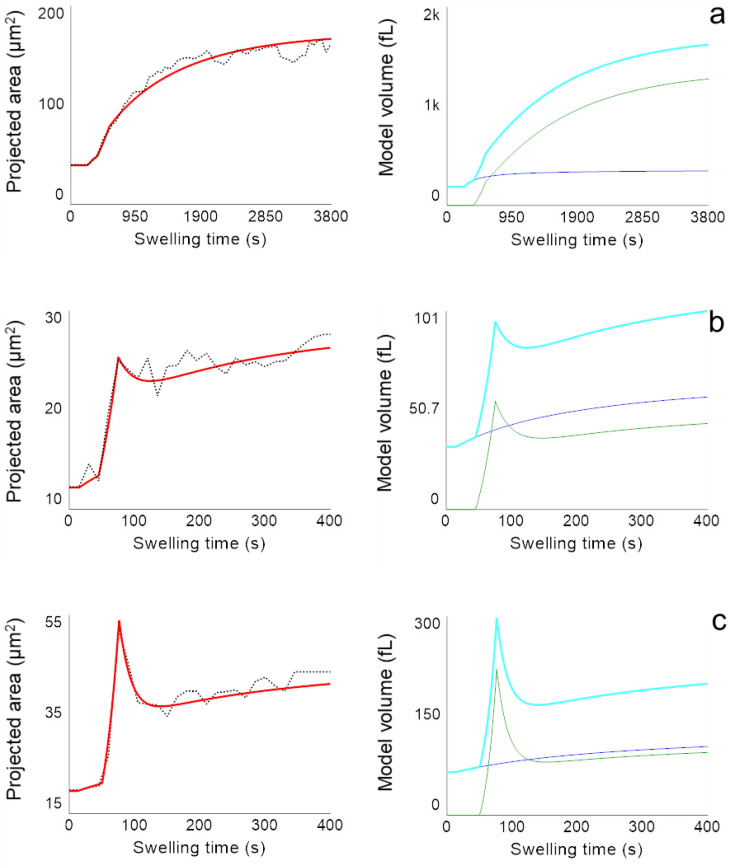
Simulation of swelling kinetics with a parallel dynamic model (red curve, **left**) based on the change of corresponding volumes (blue curve: volume I; green curve: volume II; cyan curve: total volume; **right**) for individual CMPs (**a**) at pH 11 after TGase treatment for 1 h; (**b**) at pH 14 after TGase treatment for 1 h and (**c**) at pH 14 after TGase treatment for 4 h.

Figure 5 shows the analysis of the swelling data presented in Figure 3 with the parallel dynamic model. The kinetics of the projected particle areas (red curve, Figure 5, left) are obtained by applying the hard sphere approximation to the time course of the total volume resulting from the sum of the simulated volumes I and II (cyan curve, Figure 5, right). 

From the simulation, it becomes clear that the component of cross-linked caseins (green curves in Figure 5, right) is more pronounced the higher the pH and the longer the TGase treatment time was. As the proportion of cross-links between the caseins increases with increasing treatment time, the peak also becomes more pronounced, as the comparison of Figure 5b with c shows. The pH dependence, on the other hand, can be explained by the viscoelastic material behavior of the casein network and the speed of the swelling process. The higher the pH of the exchange buffer, the faster the swelling [22].

**Figure 6:**
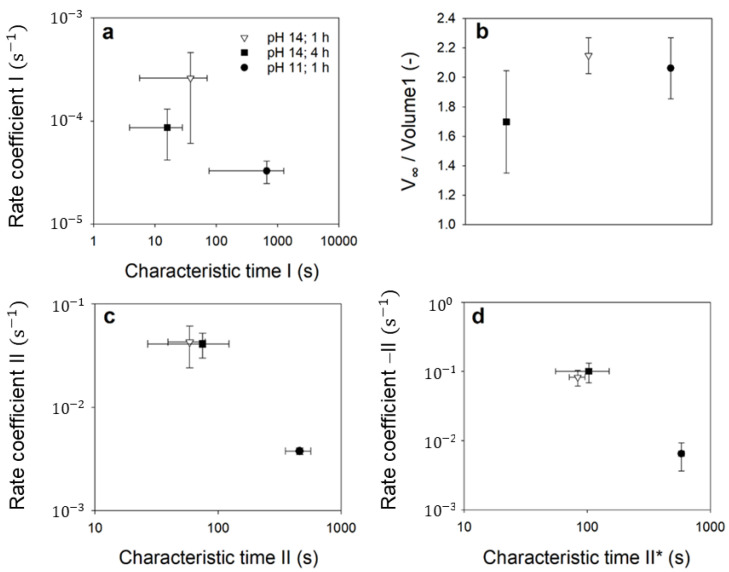
Values of the parameters, which determine the volume curves in Figure 5 via the rates of volume change: Rate coefficient versus characteristic time (**a**) and relative equilibrium swelling volume (**b**) for the uncross-linked casein and rate coefficient versus characteristic time for the swelling (**c**) and deswelling (**d**) process of the cross-linked caseins. Vertical and horizontal error bars correspond to the standard deviation.

If swelling is comparatively rapid, as at pH 14 (Figure 5b,c), overstretching of the casein network occurs and, similar to a spring, a restoring force occurs above a certain degree of swelling. The network contracts for entropic reasons and water is squeezed out of the CMPs. In contrast, if the network expands slowly, as in the case of swelling at pH 11, swelling and deswelling forces balance and the expansion of the CMPs slowly approach an equilibrium swelling value. The values of the parameters, which determine the volume curves via the rates of volume change in equations 1 and 2 are shown in Figure 6. The error bars were determined from the deviations of the model parameter values from the mean value after fitting the model to two swelling kinetics at pH 11 and pH 14 with 1 h TGase treatment and to three swelling kinetics of CMPs with 4 h TGase treatment, respectively.

**Figure 7:**
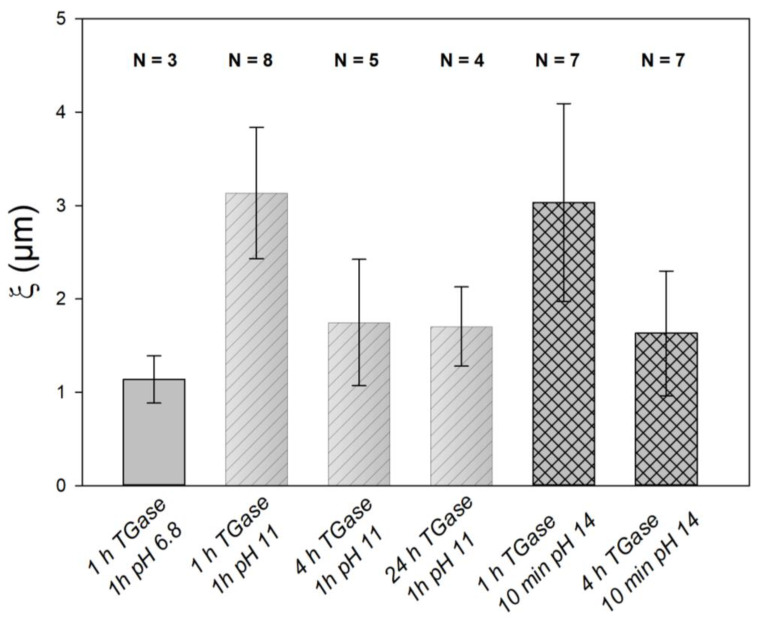
Characteristic distances of the microstructure of CMPs at different pH values and different TGase treatment times (1, 4 & 24 h) obtained by spatial correlation analysis of the gray values of line cuts through microscopic images. The error bars correspond to the standard deviation.

The rates shown in Figure 6a for CMPs treated with TGase were smaller than for untreated CMPs. The corresponding values for the untreated CMPs ranged from 0.4 to 1.5 s^−1^ at pH 14 and from 0.001 to 0.005 s^−1^ at pH 11 [22], which at pH 14 was about 3 orders of magnitude and at pH 11 2–3 orders of magnitude larger (*p* < 0.05 in each case) than the values shown here for the TGase-treated samples. In contrast, for the characteristic times at which the swelling process starts, we could not find any significant differences, between the uncross-linked CMPs from [22] and the cross-linked CMPs shown here. However, the general pH trend of the untreated CMPs, where the rates become larger and the characteristic times become smaller with increasing pH, was also observed for all the determined rates of the TGase-treated CMPs (see Figure 6a,c,d). The *p* values with *p* = 0.095 that were slightly above the significance level of 5% can be attributed to the small number of observations. In contrast to the equilibrium swelling degree for the uncross-linked casein fraction achieved, the swelling rates were indeed dependent on the pH, and thus on the charge state of the caseins. The rising negative charge with increasing pH increases the electrostatic repulsion between caseins and improves the solvent quality [43], resulting in enhanced water binding and faster expansion. However, based on the swelling data collected so far, no differences in the rates for swelling and deswelling processes were observed with respect to the treatment time with TGase. However, a longer treatment time with TGase resulted in lower equilibrium degrees of swelling or a complete loss of swelling, as shown by the comparison of the swelling curves at pH 11 in Figure 3b,c. This trend was also seen at pH 14 in the equilibrium values for the uncross-linked casein fraction (Figure 6b), although the difference here was not significant (*p* = 0.190). The lower swelling capacity can be attributed to the stronger covalent cross-linking of the CMPs, which can also be achieved by higher cross-linker concentrations instead of treatment time. Studies with different concentrations of genipin showed that stronger cross-linking due to higher concentrations of cross-linking agent resulted in smaller casein hydrogel sizes [44]. In contrast, it was reported that a higher concentration of genipin as a cross-linker [45] or a longer cross-linking treatment time with genipin [46] resulted in a lesser amount of protein or drugs released from the hydrogel, which can be attributed to a denser network structure. With swelling, the mean spacing of the building blocks ζ inside the CMPs also changes, as we have recently shown [47]. These characteristic distances can be obtained by spatial autocorrelation of the gray-scale intensity of line cuts made from microscopic images of the particles during swelling. Figure 7 shows the results of the autocorrelation analysis of freshly prepared particles, which, as indicated by the specifications on the box plot, were subjected to 1, 4, and 24 h of TGase treatment and a subsequent swelling process at pH 11 or pH 14. The unswollen state of the CMPs treated with TGase for 1 h had a correlation length of ζ = 1 µm at pH 6.8. For the same CMPs, there was an increase in correlation length to approximately ζ = 3.1 ± 0.7 µm at pH 11 and ζ = 3.0 ± 1.1 µm at pH 14 (*p* < 0.05 in both cases), which can be attributed to swelling at both pH values.

These also indicated, as the equilibrium swelling degree in Figure 6b, that the correlation length of the inner structure in TGase-treated CMPs was independent of the charge state of the caseins. This is also confirmed by the results for the CMPs with 4 h of TGase treatment. Regardless of the pH of the swelling medium, the correlation length of the internal microstructure decreased to an average value of ζ = 1.8 µm (*p* > 0.05). This value then remained unchanged even after prolonged treatment, as shown by the data at pH 11 and 24 h treatment time (*p* > 0.05).

## 3. Material and Methods

### 3.1. Materials

Sodium hydroxide (1M), hydrochloric acid (1M), sodium azide, and pectinase from *Aspergillus niger* were purchased from Merck (Merk, Darmstadt, Germany). Casein micelle (CM) concentrate powder MC88 was kindly provided by Milei GmbH Germany and citrus pectin (highly methylated pectin classic CU 201) was obtained from Herbstreith & Fox (Herbstreith & Fox GmbH & Co. KG, Neuenbürg, Germany). The product Activa WM containing microbial transglutaminase was kindly provided by Ajinomoto Foods, Hamburg, Germany. Sodium dodecyl sulfate (SDS), ultra-pure Bis-Tris, calcium chloride, and all salts (purity >99% or analytical grade) for simulated milk ultrafiltrate (SMUF) preparation were purchased from VWR, Darmstadt, Germany. Milli-Q water was obtained from our lab.

### 3.2. Preparation of Working Solutions

Simulated milk ultrafiltrate (SMUF) solution was prepared according to a procedure described by Dumpler in 2017 [48]. All salts were dissolved in milli-Q water allowing the complete dissolution of one salt before the next salt was added. Finally, pH was adjusted to 6.8 with the required amount of 1M KOH. 0.5 g/L. Sodium azide was additionally poured to SMUF in order to prevent microbial growth [31,49].

Bis-Tris buffer solution (50 mM Bis-Tris, 10 mM CaCl_2_) was prepared by adding 10.462 g Bis-Tris in a 1000 mL volumetric flask containing about 980 mL milli-Q water and stirred until dissolved. After complete dissolution, 1.11 g CaCl_2_ was added to the solution and stirred to dissolve at room temperature. Finally, the pH of the solution was adjusted to 6.8 with 1M HCl and filled to 1L with milli-Q water.

Pectin solution (2% *w*/*w*) was prepared by dissolving 1 g of highly methylated citrus pectin in 49 g Bis-Tris buffer solution (50 mM Bis-Tris, 10 mM CaCl_2_) at 80 °C with vigorous stirring for 3 h until all the pectin had dissolved and a clear solution was formed. The pectin solution was then cooled down to room temperature. Finally, the pH was adjusted to 6.8 with 1M NaOH.

Casein dispersion was prepared by dissolving MC88 powder in SMUF to obtain a final casein concentration of 7.36% (*w*/*w*). The suspension was stirred moderately for 1 h at room temperature, then overnight at 4 °C, and finally for another hour in a water bath at 37 °C, each at 150 rpm. The pH value of the casein solution was 6.7.

Pectinase solution was prepared by adding 0.47 mL pectinase (activity ≈ 800 units per mL) from *Aspergillus niger* to 10 g Bis-Tris buffer (50 mM Bis-Tris, 10 mM CaCl_2_) and mixed properly to obtain the final enzyme activity ≈ 36 units/mL. The solution was prepared immediately before adding to the film.

### 3.3. Preparation of Casein Microparticles

The CMPs were prepared by depletion flocculation induced by the addition of pectin to the casein suspension, according to the protocol described by Zhuang [15]. Pectin solution, CM dispersion and Bis-Tris buffer solution were mixed together in a ratio of 15:41:44, respectively, to obtain a mixed solution having 3% (*w*/*w*) casein and 0.3% (*w*/*w*) pectin. A lower concentration of pectin (0.3%) was used to induce a depletion flocculation reaction; where the casein-pectin attractive interaction is not dominant but the volume exclusion by pectin is responsible for the formation of casein aggregates [50]. A 3.9 g portion of this mixed dispersion was filled into a Petri dish and dried for t = 16 h overnight at T = 22 °C in a controlled environment with a relative humidity of RH = 45%. The deformable aggregates formed after the addition of pectin were then compressed and solidified by the film-drying step. For the hydrolysis of casein-pectin film, 10 mL enzyme solution was added to each Petri dish and the hydrolysis was carried out by placing the Petri dish in an Eppendorf ThermoMixer (Eppendorf AG, Germany) at T = 47 °C and shaken for t = 2 h at 300 rpm. After the hydrolysis, the turbid supernatant was carefully pipetted into a 15 mL centrifugation tube for subsequent centrifugation at 1500 g for t = 10 min at 22 °C. The resulting pellet containing the CMPs was dissolved again in BisTris buffer solution at pH 6.8 and stored at T = 4 °C in the refrigerator until further use within the next 48 h. 

### 3.4. Enzymatic Cross-Linking

For the cross-linking of the CMPs, the industrial product Activa WM, was used, containing microbial TGase with a specific activity of 81–135 U/g. The CMPs were cross-linked according to [27,31,51]. The Activa WM provided by Ajinomoto Foods, Germany was added to the sample to achieve an enzyme mass fraction of 0.155% (*w*/*w*) in the sample. The sample was incubated in a water bath at 30 °C for 1, 4 and 24 h with moderate stirring (150 rpm). The enzyme was then inactivated in the sample by heating to 70 °C for 10 min with moderate stirring and then rapidly cooled in ice water.

### 3.5. CMPs Morphology Using Line Cut Data

The internal structure of CMPs was studied using the line cuts of microscopic images. For this purpose, the microscopic images of particles with or without cross-linked and swelled at different pH conditions were taken. The image analysis software ImageJ 1.5.3 (National Institute of Health, Bethesda, MD, USA) [52] was used to extract the gray value intensities of the individual pixels along the line cuts. A detailed procedure was described elsewhere by Schulte in 2021. Briefly, a 200 × 200 pixel area around the designated particle was selected to have an identical size for the line cuts. Then, a horizontal line was drawn across the middle of the newly nominated particle for all 200-pixel distances with the help of the “Straight” selection tool. Subsequently, the gray value intensities were read along the line cut using the “Plot profile” tool. To determine the characteristic mean distance between internal granular structures of the CMPs, the line cuts were smoothed and autocorrelated using the program mathcad 15.0 (PTC Mathcad, Cambridge, MA, USA). For each sample, at least 10 particles were analyzed. 

### 3.6. Stability Experiments

For investigating the chemical stability of CMPs, the turbidity of CMP dispersions before and after the addition of sodium dodecyl sulfate (SDS) was measured at λ = 600 nm with a Lambda365 UV-VIS spectrometer from PerkinElmer, Boston, MA, USA. Two milliliters of CMPs in Bis-Tris buffer dispersion were filled into UV-Vis semi-micro cuvettes (Eppendorf AG, Hamburg, Germany) with a path length of 10 mm. For the reference sample, pure BisTris buffer solution without CMPs was used in identical cuvettes. At a wavelength of λ = 600 nm, and a gap width of 1 nm the turbidity was recorded over t = 600 s at T = RT. The turbidity was recorded once every second. After the turbidity of CMP dispersion without SDS was measured, the cuvette was taken out of the UV-Vis spectrometer and 36.6 µL of 520 mM SDS solution was added to the CMP dispersion inside the cuvette, resulting in an overall SDS concentration of 9.34 mM. The cuvette was subsequently turned upside down 2 times to ensure sufficient mixing before it was reinserted into the UV-Vis spectrometer and the measurement was started. Turbidity measurements were undertaken for CMPs that were cross-linked for t = 1 h, 4 h and 24 h and compared to CMPs without cross-linking treatment. 

### 3.7. pH-Dependent Swelling Experiments

We used the swelling setup as described by Schulte [22]. Briefly, the swelling chamber was filled with CMPs dispersion (in Bis-Tris buffer, pH 6.8) and placed under Leica DMIL LED inverted microscope (Leica Microsystems, GmbH, Wetzlar, Germany) connected with a Basler camera (Basler AG, Ahrensburg, Germany). The dispersion was allowed to stand for approx. 10 min to sediment the CMPs into the sieve holes. A PHD ULTRA™ syringe pump (Harvard Apparatus, Holliston, MA, USA) was connected with the swelling chamber by polyethylene tubes (internal diameter Ø 0.55 mm). The surrounding medium of CMPs was replaced by aqueous solution with pH 11 and 14 adjusted by NaOH. We added Thymol blue in the running solution as an indicator to prove that medium exchange was completed [21]. The pump flow rate was set at 0.05 mL per min to carry the surrounding medium (pH 11 and 14). The swelling behaviour of (N = 3) CMPs without cross-linking, (N = 2) CMPs cross-linked for t = 1 h, and (N = 2) CMPs cross-linked for t = 24 h were studied at pH 11. In addition, (N = 2) CMPs cross-linked for t = 1 h and (N = 3) CMPs cross-linked for t = 4 h were investigated at pH 14.

### 3.8. Dynamic Model and Data Analysis

A dynamic model for the swelling kinetics of the CMPs was developed based on stocks and flows using Stella software 1.6 (iseesystems.com, Lebanon, NH, accessed on 15 February 2018). Based on the model, the volume change of the CMPs results from the sum of inflows and outflows that depend on specific rates and levels of certain volume reservoirs. The system of underlying differential equations was solved using the Euler integration method with a simulation time step of 0.25 s. The transformation of the simulated volume profiles to the projected particle area was carried out using the spherical approximation. ANOVA analysis was performed to test on the basis of the obtained *p*-values whether the observed differences in the means were statistically significant.

## 4. Conclusions

Post-treatment with TGase stabilizes CMPs by preventing their SDS- and swelling-induced decomposition. While the swelling process is enhanced with increasing pH of the swelling medium as in untreated CMPs, we observe otherwise in TGase-treated CMPs that instead of decomposition, an equilibrium swelling value is reached at the end of the swelling process and that characteristic differences in the swelling curve appear. At the beginning of the kinetics, there is increased swelling, which, if it occurs rapidly as at pH 14, is followed by deswelling. Since deswelling occurs more strongly in CMPs with a long TGase treatment time, we attribute this effect to the cross-linked casein component. When the network is stretched, a restoring force occurs for entropic reasons. As a result, the elastic casein network contracts, squeezing out water and shrinking the CMPs. At pH 11, on the other hand, swelling occurs slowly without any deswelling taking place. Instead, the CMPs slowly expand, approaching the equilibrium swelling degree. All swelling kinetics of CMPs with different TGase treatment times and at different pH values can be described with a parallel dynamic model. The size changes of the CMPs can be modelled by the change of a spherical volume reservoir, which is composed of two additive subvolumes. While one partial volume represents the casein fraction cross-linked by TGases and, according to our ideas, surrounds the particles like a shell, the second partial volume stands for the uncross-linked caseins inside, which can only expand to a limited extent due to the network of cross-linked caseins. The model is also supported by the fact that both the maximum degree of swelling of this uncross-linked casein fraction and the correlation length of the microstructure is pH-independent, and that both lengths decrease with increasing TGase treatment time. Future use of angle-dependent light scattering, ultra-small angle X-ray scattering as well as high-resolution microscopic techniques also allowing nanomechanical characterisation may help in further determining whether the hypothesis of core-shell assembly of TGase-treated casein microparticles is valid [53,54,55]. Furthermore, it should be verified whether during TGase treatment inclusion of non-desirable matter such as TGase itself occurs.

## Data Availability

The datasets generated during the current study are available from the corresponding author on reasonable request.

## References

[1] Marcuello C., Chambel L., Rodrigues M.S., Ferreira L.P., Cruz M.M. (2018). Magnetotactic bacteria: Magnetism beyond magnetosomes. IEEE Trans. Nanobiosci..

[2] Kianfar E. (2021). Magnetic nanoparticles in targeted drug delivery: A review. J. Supercond. Nov. Magn..

[3] Tavares G.M., Croguennec T., Carvalho A.F., Bouhallab S. (2014). Milk proteins as encapsulation devices and delivery vehicles: Applications and trends. Trends Food Sci. Technol..

[4] Santiago L.G., Castro G.R. (2016). Novel technologies for the encapsulation of bioactive food compounds. Curr. Opin. Food Sci..

[5] Nascimento L.G.L., Casanova F., Silva N.F.N., de Carvalho Teixeira A.V.N., de Carvalho A.F. (2020). Casein-based hydrogels: A mini-review. Food Chem..

[6] Sadiq U., Gill H., Chandrapala J. (2021). Casein micelles as an emerging delivery system for bioactive food components. Foods.

[7] Holt C. (2021). A quantitative calcium phosphate nanocluster model of the casein micelle: The average size, size distribution and surface properties. Eur. Biophys. J..

[8] Fox P.F., Brodkorb A. (2008). The casein micelle: Historical aspects, current concepts and significance. Int. Dairy J..

[9] Bouchoux A., Gesan-Guiziou G., Pérez J., Cabane B. (2010). How to squeeze a sponge: Casein micelles under osmotic stress, a SAXS study. Biophys. J..

[10] Huppertz T., Gazi I., Luyten H., Nieuwenhuijse H., Alting A., Schokker E. (2017). Hydration of casein micelles and caseinates: Implications for casein micelle structure. Int. Dairy J..

[11] Dalgleish D.G. (2011). On the structural models of bovine casein micelles—review and possible improvements. Soft Matter.

[12] Sherstneva A.A., Demina T.S., Monteiro A.P., Akopova T.A., Grandfils C., Ilangala A.B. (2022). Biodegradable Microparticles for Regenerative Medicine: A State of the Art and Trends to Clinical Application. Polymers.

[13] Głąb T.K., Boratyński J. (2017). Potential of Casein as a Carrier for Biologically Active Agents. Top. Curr. Chem..

[14] Matalanis A., Decker E.A., McClements D.J. (2012). Inhibition of lipid oxidation by encapsulation of emulsion droplets within hydrogel microspheres. Food Chem..

[15] Zhuang Y., Sterr J., Kulozik U., Gebhardt R. (2015). Application of confocal Raman microscopy to investigate casein micro-particles in blend casein/pectin films. Int. J. Biol. Macromol..

[16] Maroziene A., De Kruif C.G. (2000). Interaction of pectin and casein micelles. Food Hydrocoll..

[17] Zhuang Y., Sterr J., Schulte J., Kulozik U., Gebhardt R. (2016). Casein Microparticles from Blend Films Forming Casein/α-Tocopherol Emulsion Droplets in Solution. Food Biophys..

[18] Schulte J., Pütz T., Gebhardt R. (2022). Influence of pectin and drying conditions on the structure, stability and swelling behaviour of casein microparticles. Int. Dairy J..

[19] Liu Y., Guo R. (2007). Interaction between casein and sodium dodecyl sulfate. J. Colloid Interface Sci..

[20] Lefebvre-Cases E., Gastaldi E., Tarodo De La Fuente B. (1998). Influence of chemical agents on interactions in dairy products: Effect of SDS on casein micelles. Colloids Surf. B Biointerfaces.

[21] Schulte J., Stöckermann M., Thill S., Gebhardt R. (2020). Calcium effect on the swelling behaviour and stability of casein microparticles. Int. Dairy J..

[22] Schulte J., Stöckermann M., Gebhardt R. (2020). Influence of pH on the stability and structure of single casein microparticles. Food Hydrocoll..

[23] De Kruif C.K., Anema S.G., Zhu C., Havea P., Coker C. (2015). Water holding capacity and swelling of casein hydrogels. Food Hydrocoll..

[24] Saraydın D., Karadag E., Işıkver Y., Şahiner N., Güven O. (2004). The influence of preparation methods on the swelling and network properties of acrylamide hydrogels with crosslinkers. J. Macromol. Sci. Part A.

[25] Li Q., Zhao Z. (2019). Acid and rennet-induced coagulation behavior of casein micelles with modified structure. Food Chem..

[26] Partschefeld C., Schwarzenbolz U., Richter S., Henle T. (2007). Crosslinking of casein by microbial transglutaminase and its resulting influence on the stability of micelle structure. Biotechnol. J. Healthc. Nutr. Technol..

[27] Smiddy M.A., Martin J.E., Kelly A.L., De Kruif C.G., Huppertz T. (2006). Stability of casein micelles cross-linked by transglutaminase. Int. J. Dairy Sci..

[28] Huppertz T., de Kruif C.G. (2008). Structure and stability of nanogel particles prepared by internal cross-linking of casein micelles. Int. Dairy J..

[29] Nogueira M.H., Tavares G.M., Silva N.F.N., Casanova F., Stringheta P.C., Gaucheron F., Teixeira A.V.N.C., Perrone I.T., Carvalho A.F. (2019). Physico-chemical stability of casein micelles cross-linked by transglutaminase as a function of acidic pH. Food struct..

[30] Heidebach T., Först P., Kulozik U. (2009). Microencapsulation of probiotic cells by means of rennet-gelation of milk proteins. Food Hydrocoll..

[31] Huppertz T., Smiddy M.A., de Kruif C.G. (2007). Biocompatible micro-gel particles from cross-linked casein micelles. Biomacromolecules.

[32] Bruschi M.L. (2015). Strategies to Modify the Drug Release from Pharmaceutical Systems.

[33] Fänger C., Wack H., Ulbricht M. (2006). Macroporous Poly (N-isopropylacrylamide) Hydrogels with Adjustable Size “Cut-off” for the Efficient and Reversible Immobilization of Biomacromolecules. Macromol. Biosci..

[34] Dalgleish D.G., Corredig M. (2012). The structure of the casein micelle of milk and its changes during processing. Annu. Rev. Food Sci. Technol..

[35] Heidebach T., Först P., Kulozik U. (2012). Microencapsulation of probiotic cells for food applications. Crit. Rev. Food Sci. Nutr..

[36] Gebhardt R., Pechkova E., Riekel C., Nicolini C. (2010). In situ μGISAXS: II. thaumatin crystal growth kinetic. Biophys. J..

[37] Eedara B.B., Tucker I.G., Das S.C. (2019). A STELLA simulation model for in vitro dissolution testing of respirable size particles. Sci. Rep..

[38] Vasbinder A.J., Rollema H.S., Bot A., De Kruif C.G. (2003). Gelation mechanism of milk as influenced by temperature and pH; studied by the use of transglutaminase cross-linked casein micelles. J. Dairy Sci..

[39] Zeeb B., Grossmann L., Weiss J. (2016). Accessibility of transglutaminase to induce protein crosslinking in gelled food matrices-impact of membrane structure. Food Biophys..

[40] Grossmann L., Wefers D., Bunzel M., Weiss J., Zeeb B. (2017). Accessibility of transglutaminase to induce protein crosslinking in gelled food matrices-Influence of network structure. LWT.

[41] Schott H. (1992). Swelling kinetics of polymers. J. Macromol. Sci. Part B.

[42] Thill S., Schmidt T., Jana S., Wöll D., Gebhardt R. (2022). Fine Structure and Swelling Properties of Fibers from Regenerated Rennet-treated Casein Micelles. Macromol. Mater. Eng..

[43] Vaia B., Smiddy M.A., Kelly A.L., Huppertz T. (2006). Solvent-mediated disruption of bovine casein micelles at alkaline pH. J. Agric. Food Chem..

[44] Silva N.F.N., Saint-Jalmes A., de Carvalho A.F., Gaucheron F. (2014). Development of casein microgels from cross-linking of casein micelles by genipin. Langmuir.

[45] Song F., Zhang L.M., Yang C., Yan L. (2009). Genipin-crosslinked casein hydrogels for controlled drug delivery. Int. J. Pharm..

[46] Yuan Y., Chesnutt B.M., Utturkar G., Haggard W.O., Yang Y., Ong J.L., Bumgardner J.D. (2007). The effect of cross-linking of chitosan microspheres with genipin on protein release. Carbohydrate Polymers.

[47] Schulte J., Pütz T., Gebhardt R. (2021). Statistical analysis of the swelling process of casein microparticles based on single particle measurements. Food Hydrocoll. Health.

[48] Dumpler J., Kieferle I., Wohlschläger H., Kulozik U. (2017). Milk ultrafiltrate analysis by ion chromatography and calcium activity for SMUF preparation for different scientific purposes and prediction of its supersaturation. Int. Dairy J..

[49] Marchin S., Putaux J.L., Pignon F., Léonil J. (2007). Effects of the environmental factors on the casein micelle structure studied by cryo transmission electron microscopy and small-angle x-ray scattering/ultrasmall-angle x-ray scattering. J. Chem. Phys..

[50] De Kruif C.G., Tuinier R. (2001). Polysaccharide protein interactions. Food Hydrocoll..

[51] Hinz K., Huppertz T., Kelly A.L. (2012). Susceptibility of the individual caseins in reconstituted skim milk to cross-linking by transglutaminase: Influence of temperature, pH and mineral equilibria. J. Dairy Res..

[52] Schneider C.A., Rasband W.S., Eliceiri K.W. (2012). NIH Image to ImageJ: 25 years of image analysis. Nat. Methods.

[53] Anzini P., Redoglio D., Rocco M., Masciocchi N., Ferri F. (2022). Light Scattering and Turbidimetry Techniques for the Characterization of Nanoparticles and Nanostructured Networks. Nanomaterials.

[54] Pérez-Domínguez S., Caballero-Mancebo S., Marcuello C., Martínez-Júlvez M., Medina M., Lostao A. (2022). Nanomechanical Study of Enzyme: Coenzyme Complexes: Bipartite Sites in Plastidic Ferredoxin-NADP^+^ Reductase for the Interaction with NADP^+^. Antioxidants.

[55] Velours C., Zhou J., Zecchin P., He N., Salameh M., Golinelli-Cohen M.P., Golinelli-Pimpaneau B. (2022). Determination of the Absolute Molar Mass of [Fe-S]-Containing Proteins Using Size Exclusion Chromatography-Multi-Angle Light Scattering (SEC-MALS). Biomolecules.

